# Proximal Tibiofibular Joint Arthritis Co-existing With a Medial Meniscal Tear: A Case Report

**DOI:** 10.7759/cureus.25849

**Published:** 2022-06-11

**Authors:** Eleni Pappa, Fotios Kakridonis, Ioannis A Trantos, Kyriakos Ioannidis, George Koundis, Constantine Kokoroghiannis

**Affiliations:** 1 5th Orthopaedic Department, KAT General Hospital, Athens, GRC

**Keywords:** knee pain, meniscal tear, arthritis, tibiofibular joint, knee

## Abstract

Proximal tibiofibular joint (PTFJ) arthritis is rare and, thus, not regularly considered as a source of knee pain. In this report, we present the case of a patient with posterior knee pain attributed to a medial meniscal tear rather than to a co-existing PTFJ arthritis, which was not appreciated. Based on the initial diagnosis, the patient underwent knee arthroscopy that did not alleviate his symptoms. The presence of established tibiofibular joint arthritis was diagnosed on subsequent clinical and MRI reassessment. An intra-articular corticosteroid injection settled the patient’s symptoms. The aim of this report is to raise awareness about tibiofibular joint arthritis as a possible cause of posterior or lateral knee pain.

## Introduction

The proximal tibiofibular joint (PTFJ) is a plane-type synovial joint [1]. The primary function of the PTFJ is dissipating lower leg torsional stresses and lateral tibial bending movements and transmitting axial loads in weight-bearing [2]. The articular surfaces of the PTFJ are lined with hyaline cartilage and contained within a joint capsule [3]. The joint capsule receives additional support from the anterior and posterior superior tibiofibular ligaments, lateral collateral ligaments of the knee joint, and biceps femoris [4]. The PTFJ has been regarded as “a fourth compartment to the knee” [5], as it communicates with the lateral tibiofibular space at a rate that has been reported to vary from 10% up to 63% [6].

PTFJ pathology is increasingly recognized as a cause of lateral knee pain, particularly in young adults and athletes [1,2,7]. Conditions affecting the PTFJ may be traumatic, including acute dislocation, fracture, ligament strains, and chronic subluxation. Chronic conditions such as pigmented villonodular synovitis (PVNS), neoplasm, or ganglion leading to nerve compression, have also been described [1,3,8,9]. This joint may be subject to any process that affects the knee joint proper, as is both primary and secondary arthritis [10-12]. Solitary idiopathic tibiofibular joint osteoarthritis is rarely reported especially in the absence of any systematic inflammatory disease or predisposing joint arthritis [13,14].

## Case presentation

A 51-year-old patient presented to the outpatient department complaining of long-standing posterior left knee pain, aggravated by twisting knee movements and deep knee flexion. Clinical examination was unimpressive except for the reproduction of pain upon knee flexion. On physical examination, the joint palpation did not produce any symptoms, while specialized knee tests such as the Lachman test and the McMurray test were negative for pain and instability. The extensor mechanism was intact, and no joint effusion was present. Plain radiography was unimpressive, and subsequent MRI (magnetic resonance imaging) scan revealed a posterior horn tear of the medial meniscus. The patient underwent an arthroscopic partial medial meniscectomy. On postoperative follow-up visits, the patient stated that he had no improvement of his symptoms whatsoever despite a prolonged course of physiotherapy.

**Figure 1:**
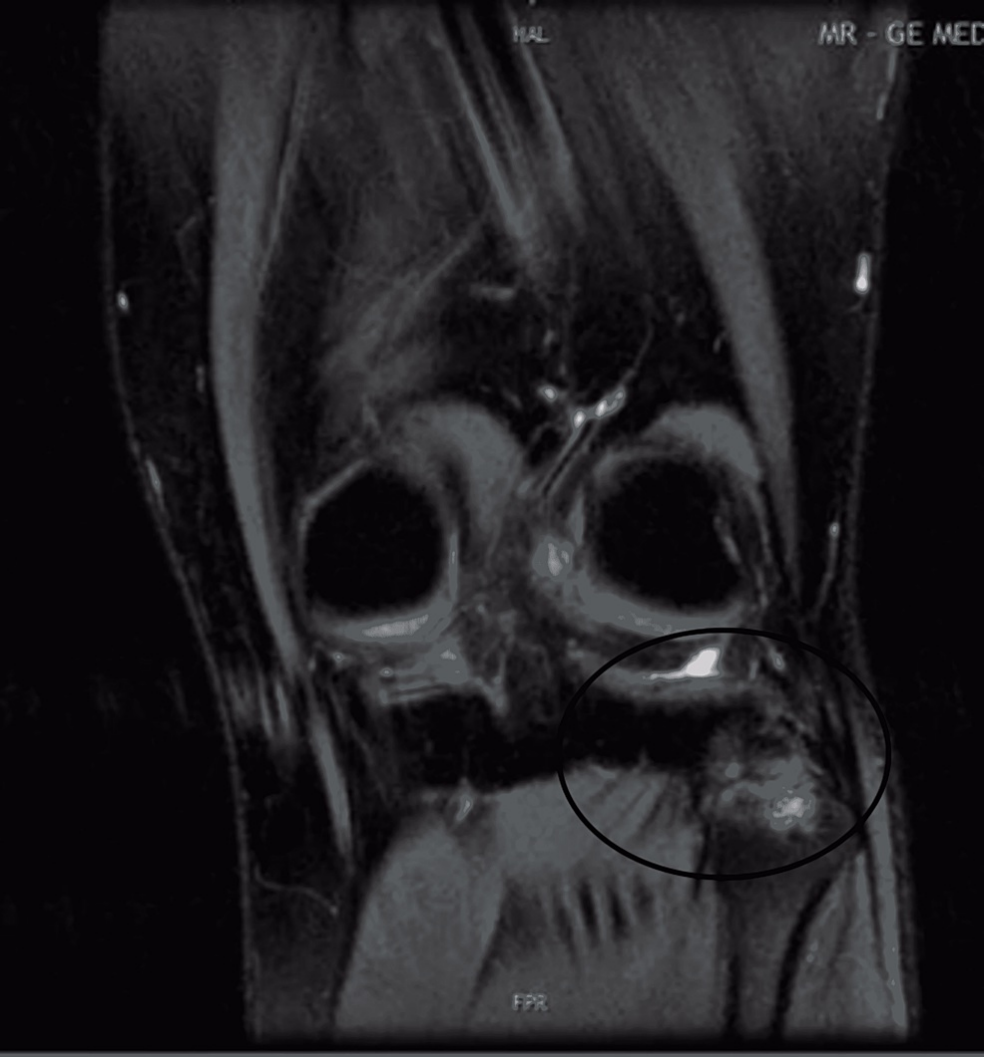
Preoperative MRI (T2 sequence) showing the osteoarthritis of the proximal tibiofibular joint

**Figure 2:**
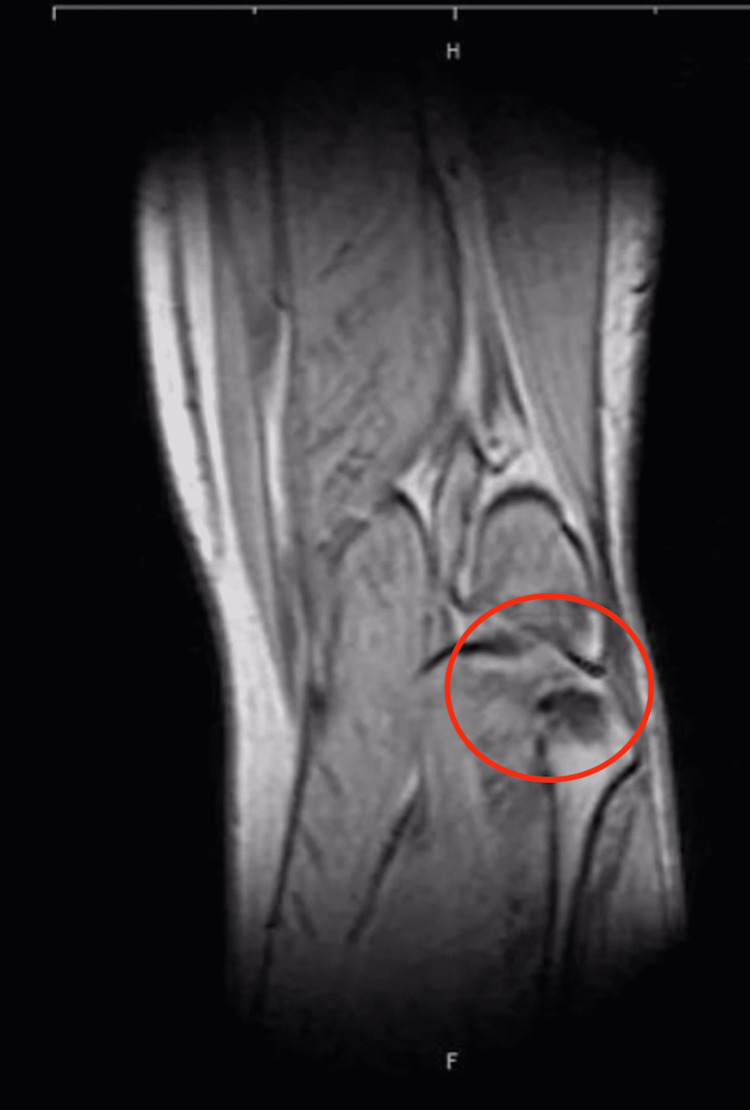
Preoperative MRI (T1 sequence) showing the osteoarthritis of the proximal tibiofibular joint

At six months postoperatively, the patient underwent a new MRI scan and was evaluated for alternative causes of pain. The new MRI revealed the presence of osteoarthritis of only the posterior part of the PTFJ with incongruity of its surfaces and subchondral edema (Figures 1, 2). Indeed, this pathology was present in the initial MRI but had not been appreciated by either the radiologist or the treating surgeon. A sonographically guided diagnostic injection via a lateral approach in the tibiofibular joint with 75mg of ropivacaine resolved the pain completely and was followed by an injection of betamethasone. Although the pain recurred after a month, it had a significantly lower intensity and allowed the patient to continue with his manual work, contemplating no further intervention.

## Discussion

Lateral knee pain is a common complaint that can be attributed to a variety of causes such as a tear of the lateral meniscus, patellofemoral pathology, iliotibial band syndrome, ligamentous injury, tumor, or infection [3,7]. Degeneration of the PTFJ can be a cause of lateral knee pain and is worth considering in the differential diagnosis. It is commonly associated with gonarthrosis in older patients [14] and can be an evasive cause of pain after a total knee replacement [15]. It is rarely reported in younger patients, being either “idiopathic” [12,14] or attributed to injury, repetitive trauma, or overuse [16]. As in other PTFJ pathologies, pain is usually lateral, but can also be anterior or posterior as in our case. It can radiate proximally or distally and have associated hamstring tightness [14,16]. Clinical examination is likely to reveal tenderness on palpation of the joint [12,16], especially on compression of the fibular head against the tibia [15].

The case presented highlights PTFJ degeneration as an alternative cause of lateral knee pain, which is worth considering. It is noteworthy that in our case, knee pain was experienced posteriorly rather than laterally, which can be explained by the selective posterior joint involvement. It can be speculated that localized incongruity was the result of injury to the posterior part of the joint. This points toward a traumatic rather than idiopathic etiology. Steroid injection was effective in alleviating the patient’s symptoms and has been also reported [17].

If symptoms recur, PTFJ arthrodesis will have to be considered. This operation has been reported to offer good pain relief [12,15]. Concomitant excision of a fibular segment to unload the distal tibiofibular joint has its advocates but is debatable.

## Conclusions

PTFJ osteoarthritis, although rare, should be considered in the differential diagnosis of lateral knee pain. Steroid injection seems to offer relief of symptoms in the short term. However, further research should be carried out in the orthopedic scientific community in order to highlight further aspects of the management of the proximal tibiofibular pathology, especially in the active patients in order to be able to return to their everyday activities.

## References

[1] Sarma A, Borgohain B, Saikia B (2015). Proximal tibiofibular joint: rendezvous with a forgotten articulation. Indian J Orthop.

[2] Forster BB, Lee JS, Kelly S, O'Dowd M, Munk PL, Andrews G, Marchinkow L (2007). Proximal tibiofibular joint: an often-forgotten cause of lateral knee pain. AJR Am J Roentgenol.

[3] Wang CR, Hing LT (2016). Proximal tibiofibular joint: an overview. J Orthop Trauma Rehabilitation.

[4] Scott J, Lee H, Barsoum W, van den Bogert AJ (2007). The effect of tibiofemoral loading on proximal tibiofibular joint motion. J Anat.

[5] Lu M, Han W, Wang K (2017). Associations between proximal tibiofibular joint (PTFJ) types and knee osteoarthritic changes in older adults. Osteoarthritis Cartilage.

[6] Ozcan O, Boya H, Oztekin HH (2009). Clinical evaluation of the proximal tibiofibular joint in knees with severe tibiofemoral primary osteoarthritis. Knee.

[7] Takai S, Yoshino N, Aso S, Hirasawa Y (2001). Symptomatic spur formation of bilateral proximal tibiofibular joints. Orthopedics.

[8] Mena H, Brautigan B, Johnson DL (2001). Split biceps femoris tendon reconstruction for proximal tibiofibular joint instability. Arthroscopy.

[9] McNamara WJ, Matson AP, Mickelson DT, Moorman CT 3rd (2018). Surgical management of proximal tibiofibular joint instability using an adjustable loop, cortical fixation device. Arthrosc Tech.

[10] Kennedy MI, Dephillipo NN, Moatshe G, Buckley PS, Bernhardson AS, LaPrade RF (2018). Proximal tibiofibular reconstruction in adolescent patients. Arthrosc Tech.

[11] Nakama GY, Gracitelli GC, de Castro Pochini a, de Souza Nery ca, Filho MC (2013). A case report: bilateral atraumatic proximal tibiofibular joint osteoarthritis. Case Rep Clin Med.

[12] Kruckeberg BM, Cinque ME, Moatshe G, Marchetti D, DePhillipo NN, Chahla J, LaPrade RF (2017). Proximal tibiofibular joint instability and treatment approaches: a systematic review of the literature. Arthroscopy.

[13] Canna SW, Chauvin NA, Burnham JM (2013). A 17 year old with isolated proximal tibiofibular joint arthritis. Pediatr Rheumatol Online J.

[14] Sundaram K, Klare CM, Moschetti WE (2018). Proximal tibiofibular osteoarthritis presenting as pain after total knee arthroplasty treated successfully with fusion of the proximal tibial-fibular joint. Arthroplast Today.

[15] Bozkurt M, Yilmaz E, Akseki D, Havitcioğlu H, Günal I (2004). The evaluation of the proximal tibiofibular joint for patients with lateral knee pain. Knee.

[16] Diaz R, Miller JE, Borg-Stein J, Kohler MJ (2016). Poster 118 ultrasound-guided proximal tibiofibular joint injection in the management of proximal tibiofibular joint arthritis and instability: a case report. PM R.

[17] Miskovsky S, Kaeding C, Weis L (2004). Proximal tibiofibular joint ganglion cysts: excision, recurrence, and joint arthrodesis. Am J Sports Med.

